# Prognostic value of ocular trauma score for open globe injuries associated with metallic intraocular foreign bodies

**DOI:** 10.1186/s12886-018-0874-3

**Published:** 2018-08-09

**Authors:** Dilek Yaşa, Zeynep Gizem Erdem, Ali Demircan, Gökhan Demir, Zeynep Alkın

**Affiliations:** Beyoğlu Eye Research and Training Hospital, Bereketzade Mah, Bereketzade Sok. No:2, Beyoğlu, İstanbul, Turkey

**Keywords:** OTS, IOFB, Trauma, Pars plana vitrectomy, PPV

## Abstract

**Background:**

The prognostic value of the ocular trauma score (OTS) in patients who underwent 23-gauge pars plana vitrectomy (23-G PPV) for surgical removal of posterior segment metallic intaocular foreign bodies (IOFB) was evaluated.

**Methods:**

Patients who underwent 23-G PPV for surgical removal of retained metallic IOFBs were retrospectively analyzed. OTS score for each patient was calculated and raw scores were converted to their corresponding OTS categories. The final VAs in study patients were compared with their respective OTS categories.

**Results:**

Twenty-five eyes from 25 patients were examined. Twenty-four (96%) of the patients were male, and the mean age was 34 ± 12 years. The time from injury to 23-G PPV was 9 ± 4 days. Fourteen (56%) patients had zone 1 trauma, eight (32%) patients had zone 2 trauma, and three (12%) patients had zone 3 trauma. Postoperative visual acuity was ≥ 20/200 in 14 (56%) of the patients and ≥ 20/40 in seven (28%) eyes. At the final visit, anatomical success was achieved in 86% of patients with retinal detachment at presentation. No statistically significant differences were found between our final VAs and OTS scores.

**Conclusion:**

OTS, which provides prognostic information after general ocular trauma, may also provide valuable prognostic information for patients who undergo 23-G PPV for the surgical removal of metallic posterior segment IOFBs.

## Background

Eyes represent only 0.27% of the body’s surface area, but they are among the most common trauma-exposed areas and can present with severe morbidity [1]. Ocular trauma is a leading cause of monocular blindness and is an important public health problem [2].

The variablity in clinical characteristics is an inherent property of trauma patients. This variability in clinical characteristics in ocular trauma patients has led several investigators to study the factors that influence both anatomical success and final post-surgical visual acuity. The ocular trauma score (OTS) is a prognostic model developed by Kuhn et al. [3]. More than 2000 cases from the United States and Hungarian Eye Injury Registries were analyzed and > 100 variables were evaluated to identify the best predictors of outcome at 6 months after injury [3]. OTS has been widely used for this purpose in open and closed globe trauma [4, 5], however, only a few studies have evaluated the prognostic value of OTS in patients with retained intraocular foreign bodies (IOFBs) [6–8].

The purpose of this study was to evaluate prognostic OTS values in patients who underwent 23-gauge pars plana vitrectomy (23-G PPV) for removal of metallic IOFBs from the posterior segment of the eyes.

## Methods

This study followed the tenets of the Declaration of Helsinki, and approval was obtained from institutional review board of Prof. Dr. N. Resat Belger Beyoglu Eye Training and Education Hospital. Medical records of patients who underwent 23-G PPV for surgical removal of retained IOFBs in the Beyoglu Eye Training and Education Hospital (BEH) during a 1-year period (January 2016–January 2017) were retrospectively analyzed. Patients who had at least 6 months of follow-up were included in the study. Demographic data of the patients, zones of injury, types of IOFBs, associated ocular pathologies, time from trauma to PPV, follow-up time, and presenting and final visual acuities (VA) were obtained from the patients’ records. Zones of injury were classified according to the Birmingham Eye Trauma Terminology System [9]. A zone I wound involves the cornea, a zone II wound extends into the anterior 5 mm of the sclera and a zone III wound involves the sclera extending more than 5 mm from limbus.

All VA examinations were performed with a back-illuminated Early Treatment Diabetic Retinopathy Study (ETDRS) chart. VAs were divided into five groups based on the OTS categories: 1.) no light perception; 2.) light perception/hand motions; 3.) 1/200 to–19/200; 4.) 20/200 to < 20/50; or 5.) ≥ 20/40. The sum of each patient’s raw points was calculated, and the numerical value was converted into the corresponding OTS score (shown in Tables 1 and 2) as described by Kuhn et al. [3]. Then, the similarity of final visual acuities by category was compared with those in the OTS study (Table 2).

**Table 1:** Ocular Trauma Score (OTS)

Variables	Raw Points
A. Initial Vision	
NLP	60
LP/HM	70
1/200–19/200	80
20/200–20/50	90
≥ 20/40	100
B. Rupture	−23
C. Endophthalmitis	−17
D. Perforating injury	−14
E. Retinal detachment	−11
F: Afferent pupillary defect	−10

*NLP* No light perception, *LP* Light perception, *HM* Hand motion

**Table 2:** Comparison of final visual acuities and OTS categorical distributions between OTS study and our series

Sum of raw points	OTS	NLP A/B	LP/HM A/B	1/200–19/200 A/B	20/200–20/50 A/B	≥20/40 A/B	*P* ^a^
0-44	1	74/0	15/0	7/100	3/0	1/0	0.116
45–65	2	27/7	26/50	18/14	15/22	15/7	0.195
66–80	3	2/0	11/0	15/0	31/50	41/50	0.856
81–91	4	1/0	2/0	3/0	22/33	73/67	0.733
92–100	5	0/0	1/0	1/0	57/0	94/0	N/A

*OTS* Ocular Trauma Score, *NLP* No light perception, *LP* light perception, *HM* Hand motion, *A* OTS Study results (%), *B* Our study results (%)

^a^Chi-square test

### Surgical technique

All eyes underwent standard 23-G PPV using the Constellation Surgical Vitrectomy System (Alcon Laboratories Inc., Fort Worth, TX, USA). Pars plana lensectomy or phacoemulsification was performed in eyes with lens opacity. In eyes with retinal detachment (RD), perfluorocarbon liquids were used to attach the retina, and retinal breaks were treated with endophotocoagulation. To remove the IOFBs, one of the sclerotomies were enlarged, and IOFBs were removed using intraocular forceps. Air, sulfur hexafluoride (SF6), perfluoropropane (C3F8), or 1000 cSt silicone oil was used at the end of the surgery as an internal tamponade. Sclerotomies were sutured at the end of the surgery. In eyes with RD, anatomical success was defined as total retinal attachment at the final visit.

### Statistical analysis

Continuous data are presented as means and standard deviation while categorical data are presented as percentages. Chi square test was used for comparison of categorical distributions of final visual acuities and OTS scores between OTS study and our series. Mann-Whitney U test was performed for the comparison of final visual acuities between the patients with and without RD. Additionally, Fisher’s exact test was used to compare the categorical distribution of VAs in patients with and without RD. A two-tailed *p*-value < 0.05 was considered statistically significant.

## Results

**Fig. 1:**
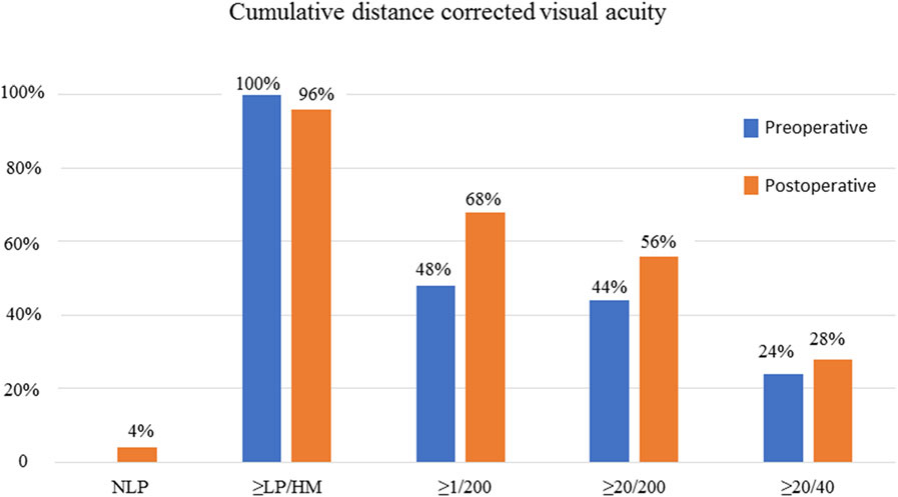
Cumulative distance-corrected visual acuity

Twenty-five eyes from 25 patients were included in the study. Twenty-four (96%) of the patients were male, and the mean age was 34 ± 12 years (min:12, max: 64, median 37). The mean follow-up time was 10 ± 5 months (min: 6, max: 21, median 6). Presenting and final VAs of each patient are shown in Table 3. Cumulative VAs are presented in Fig. 1. At the final visit VA was 20/200, or better in 56% and 20/40, or better in 28% of the eyes. Cumulative VAs in patients with or without a RD are shown in Table 4. Our hospital is a tertiary referral eye hospital and most of our patients are referred from rural areas of Turkey or from other hospitals. Accordingly, 7 (28%) eyes had RD at presentation, and although anatomical success was achieved in 6 (85.7%) of these, only 14.3% of the eyes had a final VA of 20/40. Cumulative VAs were better in eyes without RD. However, the difference was not statistically significant (Table 4). Work-related trauma (96% of eyes) was the most common cause of the injury, which was followed by car accidents (4% of eyes). None of the patients was wearing protective safety glasses during trauma. Mechanism of injury are presented in Table 5.

**Table 3:** Presenting and final visual acuities and OTS of individual patients

No	Age	Gender	OTS	OTS Category	VA (admission)	VA (final)
1	40–50	Male	28	1	LP	20/1500
2	40–50	Male	56	2	HM	20/100
3	40–50	Male	56	2	HM	HM
4	20–30	Male	56	2	HM	20/1500
5	60–70	Male	56	2	LP	HM
6	20–30	Male	56	2	LP	20/400
7	10–20	Male	56	2	LP	HM
8	30–40	Male	56	2	LP	HM
9	30–40	Male	56	2	LP	HM
10	30–40	Male	65	2	20/50	20/32
11	30–40	Male	45	2	HM	HM
12	10–20	Female	45	2	HM	HM
13	40–50	Male	45	2	LP	20/200
14	30–40	Male	55	2	5/200	20/100
15	10–20	Male	45	2	LP	NLP
16	40–50	Male	76	3	20/63	20/200
17	10–20	Male	76	3	0.16	20/20
18	30–40	Male	76	3	20/50	20/200
19	40–50	Male	76	3	20/50	20/32
20	20–30	Male	86	4	20/32	20/63
21	30–40	Male	86	4	20/40	20/32
22	40–50	Male	86	4	20/25	20/25
23	20–30	Male	86	4	20/20	20/20
24	30–40	Male	86	4	20/20	20/20
25	20–30	Male	86	4	20/32	20/63

*OTS* Ocular trauma score, *VA* Visual acuity (Snellen), *LP* Light perception, *NLP* No light perception, *HM* Hand motion

**Table 4:** Visual acuities in eyes with and without retinal detachment

	Eyes with RD *n* = 7	Eyes without RD *n* = 18	*p*
VA (Mean ± SD, logMAR)	1.64 ± 1.24	1.26 ± 1.27	0.398*
VA ≥ LP/HM (%)	85.7	100	0.280**
VA ≥ 1/200 (%)	57.1	72.7	0.640**
VA ≥ 20/200 (%)	42.9	61.1	0.656**
VA ≥ 20/40 (%)	14.3	33.3	0.626**

*HM* Hand motions, *LP* Light perception, *n* Number of eyes, *SD* Standard deviation, *RD* Retinal detachment, *VA* Visual acuity

*: Mann Whitney U test, two-tailed *p* value

**: Fisher’s Exact Test, two tailed *p* value

**Table 5:** Mechanism of injury

	n (%)
Hammering	11 (44)
Chisseling	3 (12)
Drilling	4 (16)
Cutting metal	6 (24)
Car crash	1 (4)

*n* Number of eyes

There were 14 (56%), eight (32%), and three (12%) cases of zones 1, 2, and 3, respectively. The mean time from injury to 23-G PPV was 9 ± 4 days. Associated ocular pathologies are shown in Table 6 and injured intraocular structures are shown in Table 7. A metallic IOFB was found in all eyes. Posterior segment IOFBs were observed in 10 (40%) eyes. In the remaining 4 (16%) eyes, IOFB located in the vitreous cavity, 9 (36%) in the peripheral retina, and 2 (8%) nasal relative to the optic disc in the posterior pole of the eye. A total of 13 (52%) eyes had cataract at the presentation. Of the 6 eyes with visually significant cataracts, 3 underwent pars plana lensectomy without intraocular implantation and 3 underwent phacoemulsification and intraocular lens implantation. One eye with pars plana vitrectomy underwent intraocular lens placement with scleral fixation 6 months after the first surgery. In the remaining 2 eyes, lens placement was not considered because of the poor visual prognosis. All IOFBs were successfully removed, and anatomical success was achieved at the final visit in 96% of 25 eyes with or without RD at presentation.

**Table 6:** Associated ocular pathologies

Ocular Pathology	n (%)
Cyclodialysis	3 (12)
Iridodialysis	1 (4)
Traumatic Cataract	13 (52)
Intravitreal hemorrhage	8 (32)
Choroidal detachment	1 (4)
Retinal detachment	7 (28)
Endophtalmitis	1 (4)

*n* Number of eyes

**Table 7:** Injured intraocular structures

	n (%)
Zone 1	14 (56)
Zone 2	8 (32)
Zone 3	3 (12)
Iris/ciliary body	4 (16)
Crystalline lens	13 (52)
Macula	2 (8)
Peripheric retina	9 (36)
Sclera	11 (44)

*n* Number of eyes

The most common postoperative complication was an increase in intraocular pressure (12%), but it was controlled with medical treatment in all patients. One eye with RD at baseline had macula-off RD and no light perception at the final visit. In one patient, who had a culture that was negative for endophthalmitis at presentation, recurrent RD occurred, but anatomical success was achieved after the second 23-G PPV. Air was used as an internal tamponade in 8 (32%) eyes, SF6 in 2 (8%) eyes, C3F8 in 3 (12%) eyes, and silicone oil in 12 (48%) eyes. One eye with RD at the presentation treated with PPV and silicone oil injection and 1 eye without RD treated with PPV and C3F8 injection showed RD during follow-up. They underwent second PPV to achieve retinal reattachment. At the final visit, retina was still attached in those 2 patients. Silicone oil was removed in 12 eyes in the mean time of 5.7 months after PPV. In the remained one eye with cyclodialysis at the presentation, silicone oil was not removed because of the chronic hypotonia. Final VAs based on OTS categories and the OTS scores are presented in Table 2.

All patients were administered systemic antibiotics (moxifloxacin) until IOFB removal with PPV. Only 3 patients were given intravitreal antibiotics (vancomycin and ceftazidime), solely based on surgeon preference, not signs of endophthalmitis. Topical antibiotics were applied as an appropriate adjuvant to intravitreal and systemic antibiotic use once the wound was closed following PPV.

## Discussion

IOFBs may be associated with up to 40% of open globe injuries especially with respect to work related injuries [10, 11]. Male dominance and the peak patient age in the young to middle-aged group in our study was in agreement with reports in the literature in which young to middle-aged working males are at the highest risk for ocular trauma.

Greven et al. reported final VAs of 20/40 of more were obtained in 71% of IOFB-associated open globe injuries [12]. Although their results are superior to those associated with the general functional outcomes in other types of open globe trauma, other investigators have reported that functional success is not more common in eyes with IOFB [4]. Ahmadieh et al. [13] found that IOFB was a poor predictor of visual outcome. These conflicting findings probably result from the fact that the patients with IOFB are a heterogeneous group with different clinical characteristics that are dependent on the nature of the injury and the foreign body.

Anatomical and visual outcomes, as well as postoperative complications in this study were comparable to those reported in the literature [4–6]. The final visual acuity of eyes with RD was lower when compared to eyes without a RD. However, it did not reach the level of statistical significance. The reason for the lack of statistically significant difference between the groups (eyes with RD and eyes without RD) may be the low number of patients (only seven patients had RD), the complex nature of trauma, heterogeneity of this patient group, and other factors limiting functional success in patients who do not have RD at presentation.

OTS has been widely in open and closed globe trauma [4, 5, 13–15]. However, it had been validated in only a few subgroups of patients with IOFBs. Unal et al. [6] reported the prognostic value of OTS in cases of deadly weapon-related open-globe injuries with IOFB, Purtskhvanidze et al. [7] in rotating wire brush injuries with IOFB, and Zhu et al. [8] in siderosis bulbi with retained IOFB. Although all of them were associated with IOFBs, the mechanism of injury, presenting clinical characteristics, and distribution of OTS scores were very different among these studies. On one hand, 40% of the patients in the Purtskhvanidze et al. [7] study were OTS 5, whereas none of the patients in the Unal et al. [6] study were OTS 5. Results from Unal et al. [6] were similar to those in this OTS study, except for OTS category 2; results from Purtskhvanidze et al. [7] were similar to those in this OTS study, except for OTS category 1. Results from the study by Zhu et al. [8] were similar to those in this OTS study in all categories.

Our study group was different from the others in the literature in that we only included cases of metallic posterior segment IOFBs that required PPV. However, we obtained similar results and showed that OTS can successfully predict the functional outcome after successful removal of the IOFB with 23-G PPV. We had no patient in OTS category 5, but the final VAs were similar to the OTS categories.

One limitation of our study was the low number of patients and retrospective study design. Also, we evaluated pediatric and adult patients together due to the low number of patients. In addition, there may be a geographical bias that is inherent to trauma studies. This fact underlies the need for a National Eye Registry and multicenter studies for trauma patients in Turkey. However, the relatively homogeneous patient group in this study showed that OTS was useful for this special subgroup of IOFB.

## Conclusions

In conclusion, we found that OTS, which provides prognostic information after general ocular trauma, may also provide reliable prognostic information on patients who undergo 23-G PPV for the surgical removal of metallic posterior segment IOFBs. OTS may offer the possibility of approximation of the functional result in these patients before the surgery.

## Abbreviations

23 G PPV: 23-gauge pars plana vitrectomy
C3F8: Perfluoropropane
HM: Hand motion
IOFB: Intraocular foreign body
IOP: Intraocular pressure
LP: Light perception
OTS: Ocular trauma score
PPL: Pars plana lensectomy
RD: Retinal detachment
SF6: Sulfur hexafluoride
VA: Visual acuity
